# Plasma-Activated Water Improve Wound Healing in Diabetic Rats by Influencing the Inflammatory and Remodelling Phase

**DOI:** 10.3390/ijms26031265

**Published:** 2025-01-31

**Authors:** Jovana Rajić, Nevena Grdović, Anđelija Marković, Nikola Škoro, Svetlana Dinić, Aleksandra Uskoković, Jelena Arambašić Jovanović, Marija Đorđević, Ana Sarić, Melita Vidaković, Nevena Puač, Mirjana Mihailović

**Affiliations:** 1 Department of Molecular Biology, Institute for Biological Research “Siniša Stanković”, National Institute of Republic of Serbia, University of Belgrade, Bulevar despota Stefana 142, 11108 Belgrade, Serbia; nevenag@ibiss.bg.ac.rs (N.G.); sdinic@ibiss.bg.ac.rs (S.D.); auskokovic@ibiss.bg.ac.rs (A.U.); jelena.arambasic@ibiss.bg.ac.rs (J.A.J.); marija.sinadinovic@ibiss.bg.ac.rs (M.Đ.); ana.saric@ibiss.bg.ac.rs (A.S.); melita@ibiss.bg.ac.rs (M.V.); mista@ibiss.bg.ac.rs (M.M.); 2 Center for Non-Equilibrium Processes, Institute of Physics, National Institute of Republic of Serbia, University of Belgrade, Pregrevica 118, 11080 Belgrade, Serbia; andjelija@ipb.ac.rs (A.M.); nskoro@ipb.ac.rs (N.Š.)

**Keywords:** plasma-activated water, diabetes, wound healing, inflammation, extracellular matrix remodelling, collagen, matrix metalloproteinase-9

## Abstract

Diabetic foot ulcers have an enormous impact on patients’ quality of life and represent a major economic burden. The cause is delayed and incomplete wound healing due to hyperglycemia, reduced blood flow, infections, oxidative stress and chronic inflammation. Plasma-activated water (PAW) is emerging as a new therapeutic approach in wound treatment, as it has many of the advantages of cold atmospheric plasma but is easier to apply, thus allowing for widespread use. The aim of this study was to investigate the potential of PAW to improve wound healing in diabetic rats, with a focus on uncovering the underlying mechanisms. Two full-thickness wounds in control and diabetic animals were treated with PAW, and healing was monitored for 15 days at five time points. PAW improved wound healing in diabetic rats and mainly affected the inflammatory phase of wound healing. Application of PAW decreased the number of inflammatory cells, myeloperoxidase (MPO) and N-acetyl-b-D-glycosaminidase (NAG) activity, as well as the mRNA expression of proinflammatory genes in diabetic rats. Ten days after injury, PAW treatment increased collagen deposition in the diabetic animals by almost 10% without affecting collagen mRNA expression, and this is in correlation with a decrease in the *Mmp-9*/*Timp-1* ratio. In conclusion, PAW treatment affects wound healing by reducing the inflammatory response and influencing extracellular matrix turnover, suggesting that it has great potential to accelerate the healing of diabetic wounds.

## 1. Introduction

Wound healing is a highly complex and dynamic process that occurs through well-organized communication between different cell types and the extracellular matrix (ECM) and is mediated by numerous growth factors and cytokines [1,2]. Under physiological conditions, wound healing is a highly efficient process consisting of four continuous and overlapping phases: hemostasis, inflammation, proliferation and remodelling [3]. Hemostasis is characterized by the aggregation of platelets and the formation of a fibrin clot, followed by the recruitment of inflammatory cells [4]. The inflammatory phase begins with the infiltration of neutrophils, followed by the migration of monocytes and lymphocytes into the wound bed. The purpose of inflammation is to clear debris, prevent possible infection and initiate the proliferative phase through the action of growth factors and cytokines, whose presence induces the proliferation of keratinocytes and fibroblasts. In the proliferative phase, the injured area fills with new tissue through which keratinocytes migrate from the wound edges to restore the damaged epidermis. Eventually, the skin restores its structural and functional properties by remodelling the underlying ECM and reducing the blood supply [5,6,7,8].

In diabetic patients, wound healing is delayed and often incomplete, which is characterized by the loss of coordination between the processes that govern wound healing [9]. The resulting diabetic chronic wounds usually occur on the patient’s feet and, in addition to their direct impact on quality of life, also represent an enormous economic burden [10,11]. Foot ulcers occur in up to 25% of patients with long-term diabetes [12] and are the most common cause of limb amputations [13]. The underlying pathology of diabetic foot ulceration consists of a combination of reduced blood flow, infection, oxidative stress and chronic inflammation, all of which are associated with diabetes and impede proper wound healing [14,15,16]. Standard wound management in diabetes includes a range of treatments aimed at removing devitalized tissue, controlling infection and keeping the wound moist and vascularized [17]. The overuse of antibiotics and the resulting resistance, as well as the difficulty in controlling inflammation, make diabetic wound management insufficiently efficient [18]. Therefore, there is a constant need for innovative interventions that meet the therapeutic requirements at different stages of chronic wound healing. As technology advances, active multifunctional wound dressings that can sequentially deliver bioactive substances, such as films, nanofiber membranes and hydrogels, have been developed and have shown promise as therapeutic agents for wound healing in diabetic patients [19,20,21].

In recent years, the application of cold atmospheric plasma (CAP) has been considered a promising approach for wound healing [7,22]. CAP is a partially ionized gas that exists at atmospheric pressure and room-ambient conditions. The temperature of CAP is below 40 °C, which makes it suitable for the treatment of cells and tissue. The biological activity of CAP is attributed to the rich plasma chemistry, particularly the reactive oxygen and nitrogen species (RONS) [23,24], the concentration of which can be adjusted by the choice of working gas mixtures, the type of plasma source and the plasma parameters applied [25]. The RONS present in CAP can cross a cell membrane and enter the cell interior through a membrane channel, a passive diffusion process or pores created by lipid oxidation. An appropriate intensity of plasma generates a physiological level of RONS, which are able to promote the cell proliferation of endothelial cells, keratinocytes and fibroblasts, and thus the wound healing process. In addition to the proliferation-promoting effects, RONS induces changes in the expression of a number of cytokines and growth factors that have a decisive influence on the effectiveness of wound healing [26]. Treatment with CAP has been shown to be beneficial for chronic wound healing due to its well-documented antimicrobial activity [27] and its ability to stimulate the recruitment of inflammatory cells and the migration and proliferation of keratinocytes and fibroblasts [7,28,29,30]. Treatments have recently evolved into in vivo studies, as well as clinical trials, focusing on the detailed investigation of the healing process [31,32]. Considering that the currently used CAP systems are bulky, limiting their wide application, increasing evidence of the comparable efficacy of plasma-activated water (PAW) suggests that PAW is a potential solution, especially for disabled diabetic patients. The biological activity of PAW is attributed to the synergistic effect of long- and short-lived RONS [33]. PAW has been shown to accelerate wound healing [27], but the mechanisms, apart from the antimicrobial properties, have not been studied in detail. Here, the potential of PAW to improve wound healing in diabetic rats is investigated with particular reference to the progression of the different phases of the wound healing process.

## 2. Results

### 2.1. Improved Wound Closure in Diabetic Rats After PAW Treatment

**Figure 1:**
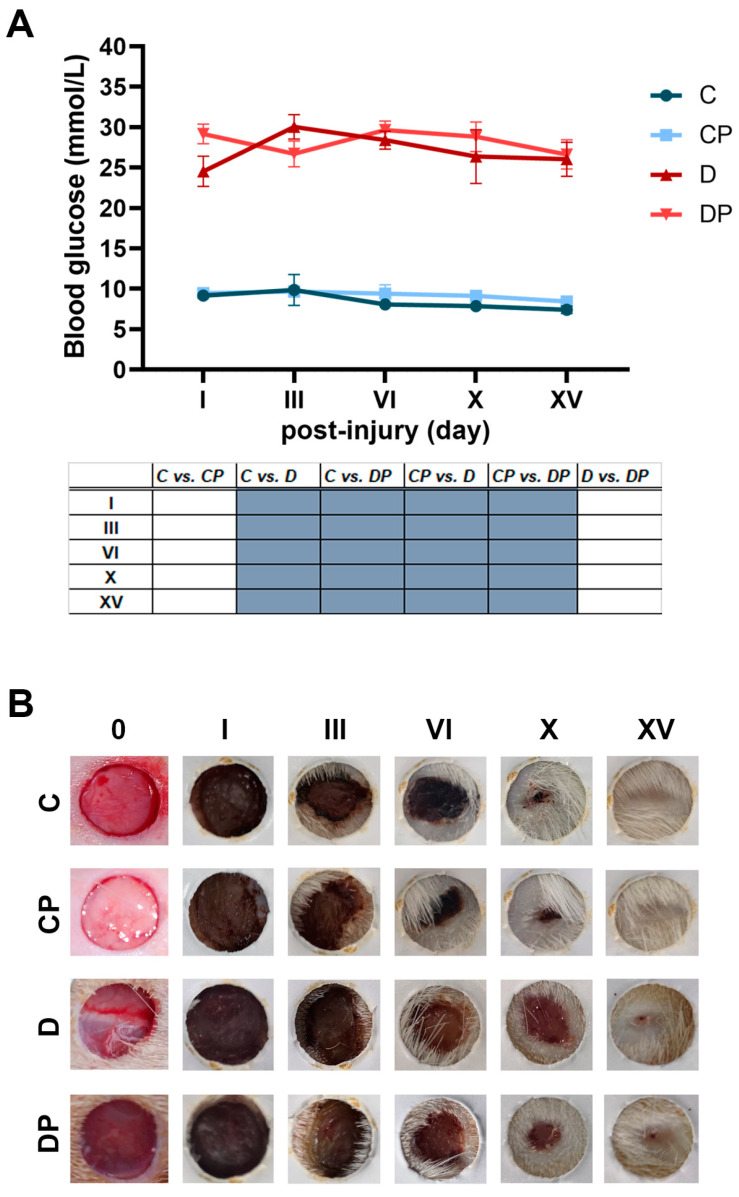

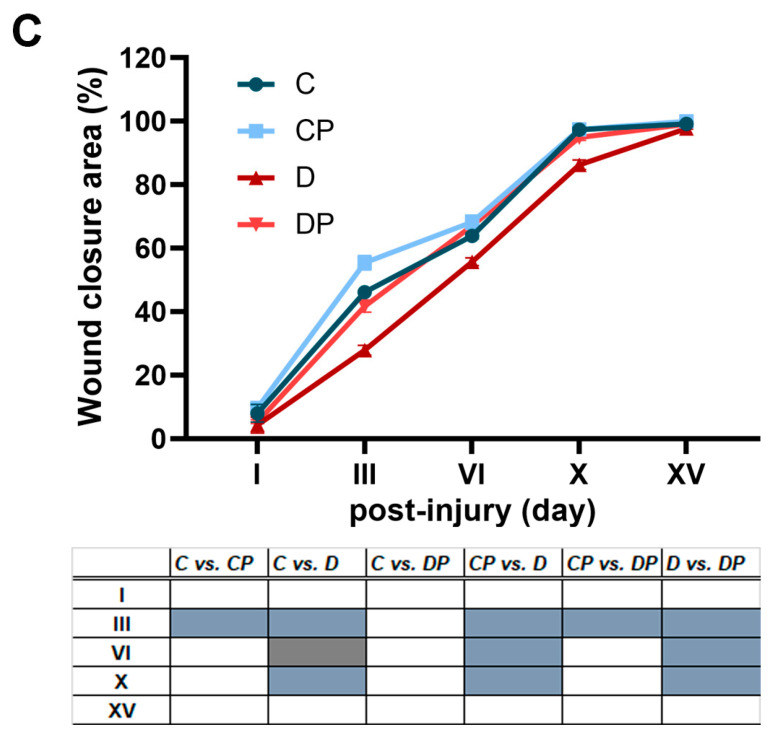
**The effect of PAW treatment on the progression of wound healing in control and diabetic rats**. (**A**) Blood glucose levels during the treatment period. (**B**) Representative photographs of the full-thickness skin wounds with or without PAW treatment in control and diabetic rats. (**C**) The wound closure area (%) during the healing process in control and diabetic rats with or without PAW treatment. C–Control non-diabetic group (*n* = 3); CP–PAW-treated control non-diabetic group (*n* = 3); D–diabetic group (*n* = 3); DP–PAW-treated diabetic group (*n* = 3). Values are expressed as means ± SEM, and the results of the ordinary two-way ANOVA and Tukey’s post hoc tests are presented in the tables below the graphs: dark grey *p* ≤ 0.01; blue *p* ≤ 0.001.

To evaluate the effects of PAW treatment on diabetic wound healing, we used a full-thickness wound model in rats and followed different aspects of wound healing at five time points (day I, III, VI, X, XV). PAW treatment had no effect on blood glucose levels (Figure 1A) and caused no changes in wound healing after day I (Figure 1B,C). In contrast to day I, monitoring of wound closure kinetics showed that PAW treatment of the diabetic rats significantly improved wound closure on days III, VI and X, bringing it to the level of the control groups. PAW treatment did not affect wound closure in the control animals, except on day III post-injury when a significant increase in CP was observed compared to the C group.

**Figure 2:**
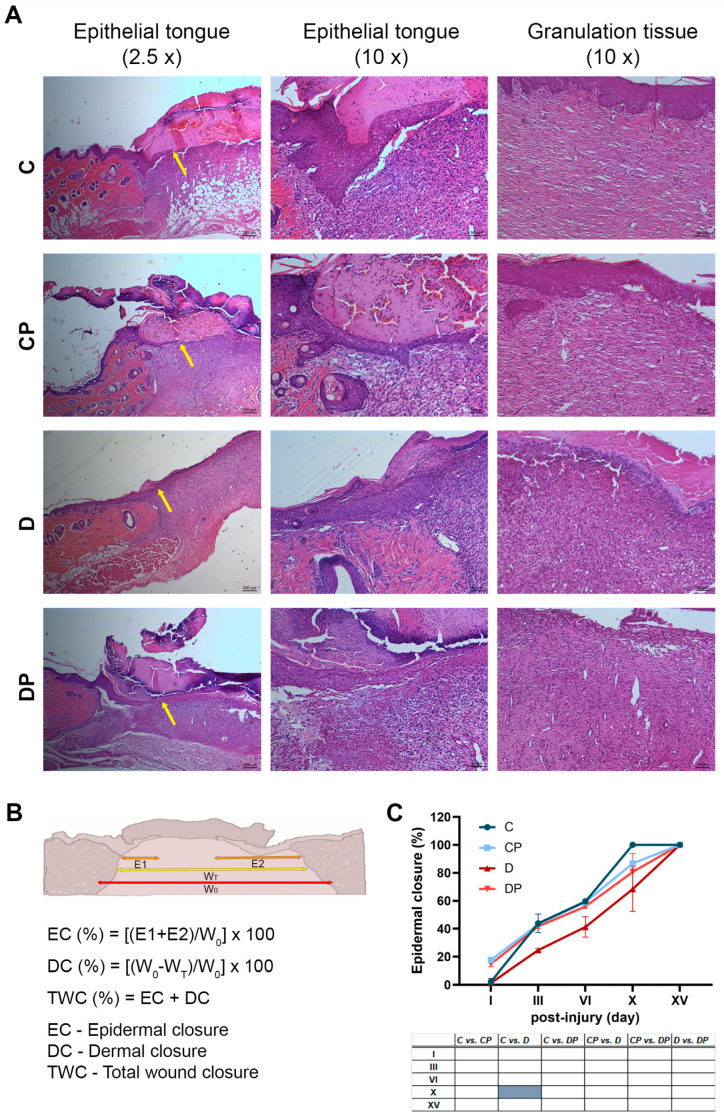

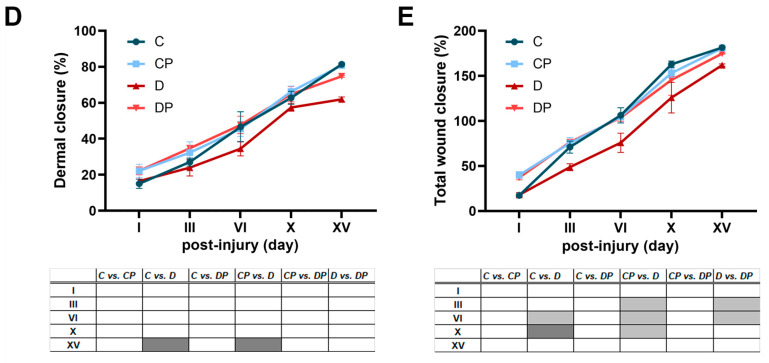
**Histological evaluation of wound healing in control and diabetic rats with or without PAW treatment**. (**A**) Haematoxylin–eosin staining of wounds with or without PAW treatment in control and diabetic rats. The progression of re-epithelialization at day VI is marked by yellow arrows (magnifications 2.5× and 10×), while the structure of granulation tissue is presented on day X after the injury (magnification 10×). (**B**) A schematic presentation of histologic measurements and formulas used for the calculation of epidermal closure (**C**), dermal closure (**D**) and total wound closure (**E**). C–Control non-diabetic group (*n* = 3); CP–PAW-treated control non-diabetic group (*n* = 3); D–diabetic group (*n* = 3); DP–PAW-treated diabetic group (*n* = 3). Values are expressed as means ± SEM, and the results of the ordinary two-way ANOVA and Tukey’s post hoc tests are presented in the tables below the graphs: light grey *p* ≤ 0.05; dark grey *p* ≤ 0.01; blue *p* ≤ 0.001.

Comparable results were obtained after measuring complete wound closure on H&E-stained skin sections (Figure 2). As shown in the representative images, the results of H&E staining revealed a pronounced epithelial tongue spreading towards the wound surface in groups C, CP and DP (Figure 2A, yellow arrows), as well as the better maturation of granulation tissue in these groups. Quantification revealed that total wound closure was delayed in group D compared to all other groups on days III, VI and X (Figure 2E). In addition, a histologic analysis showed that the PAW-treated groups had better healing at the beginning of the healing process (day I) compared to the non-treated groups, as epidermal and dermal wound closure increased by 15.5% and 7.2%, respectively, in the CP group compared to the C group and by 13.5% and 5.7%, respectively, in the DP group compared to the D group (Figure 2C,D). This improvement in healing on day I could not be determined by simply photographing and measuring the wound closure area (Figure 1B,C). Histologic analysis also showed slightly better overall wound closure in the DP group compared to the D group on day XV post-injury (Figure 2D,E). This can be attributed to the 12.8% difference in dermal closure between these two groups, considering that the epidermis was completely closed in all groups on this day. It suggest that epidermal and dermal wound closure follow slightly different kinetics and that PAW treatment of diabetic animals improves the healing of cutaneous wounds, particularly affecting processes in granulation tissue.

### 2.2. PAW Treatment Attenuates Inflammatory Response in Diabetic Rats

**Figure 3:**
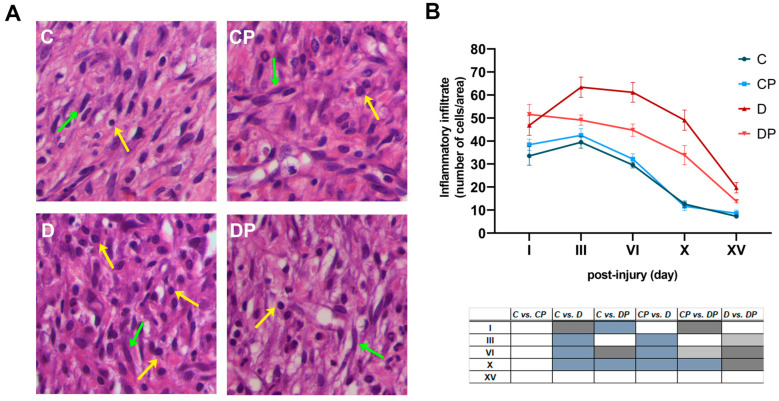

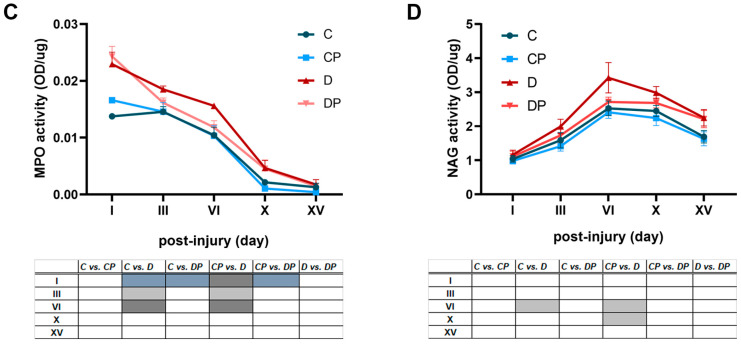
**The effect of PAW treatment on inflammatory response during wound healing in control and diabetic rats**. (**A**) Haematoxylin–eosin staining of granulation tissue of wounds with or without PAW treatment in control and diabetic rats at day VI (magnification 40×). Inflammatory cells are indicated by yellow and fibroblasts by green arrows. (**B**) The number of inflammatory cells counted per area. (**C**) Biochemical quantification of myeloperoxidase (MPO) activity as a measure of neutrophil infiltrate. (**D**) Biochemical quantification of N-acetylglucosaminidase (NAG) as a measure of macrophage infiltration. C–Control non-diabetic group (*n* = 3); CP–PAW-treated control non-diabetic group (*n* = 3); D–diabetic group (*n* = 3); DP–PAW-treated diabetic group (*n* = 3). Values are expressed as means ± SEM, and the results of the ordinary two-way ANOVA and Tukey’s post hoc tests are presented in the tables below the graphs: light grey *p* ≤ 0.05; dark grey *p* ≤ 0.01; blue *p* ≤ 0.001.

The effect of PAW treatment on the progression of the inflammatory stage of wound healing was investigated using H&E staining (Figure 3A). Quantification of the inflammatory infiltrate showed a small initial increase in inflammatory cells in the PAW-treated groups compared to the control and diabetic groups (Figure 3B). During the entire observation period, PAW treatment had no effect on the number of inflammatory cells in the control animals. In contrast, PAW treatment led to a significant decrease in the inflammatory infiltrate in the diabetic animals compared to the untreated diabetic group on days III, VI and X after injury.

Since a simple counting of the inflammatory infiltrate could not distinguish between different types of inflammatory cells, we performed MPO and NAG activity assays to obtain more specific results. The MPO assay showed that neutrophil activity was highest at the beginning of the healing process and gradually decreased over time (Figure 3C). On day I after injury, MPO activity was higher in the PAW-treated groups than in the non-PAW-treated groups. The NAG activity assay showed the peak of macrophage activity on day VI post-injury in all groups (Figure 3D). Interestingly, NAG activity decreased to control levels on days III, VI and X in the diabetes group treated with PAW. These results suggest that PAW treatment of diabetic animals has the potential to influence the establishment, progression and resolution of the inflammation in a way that is more similar to the course of the inflammatory phase of the control animals.

### 2.3. Increased Collagen Deposition in Diabetic Rats After PAW Treatment

**Figure 4:**
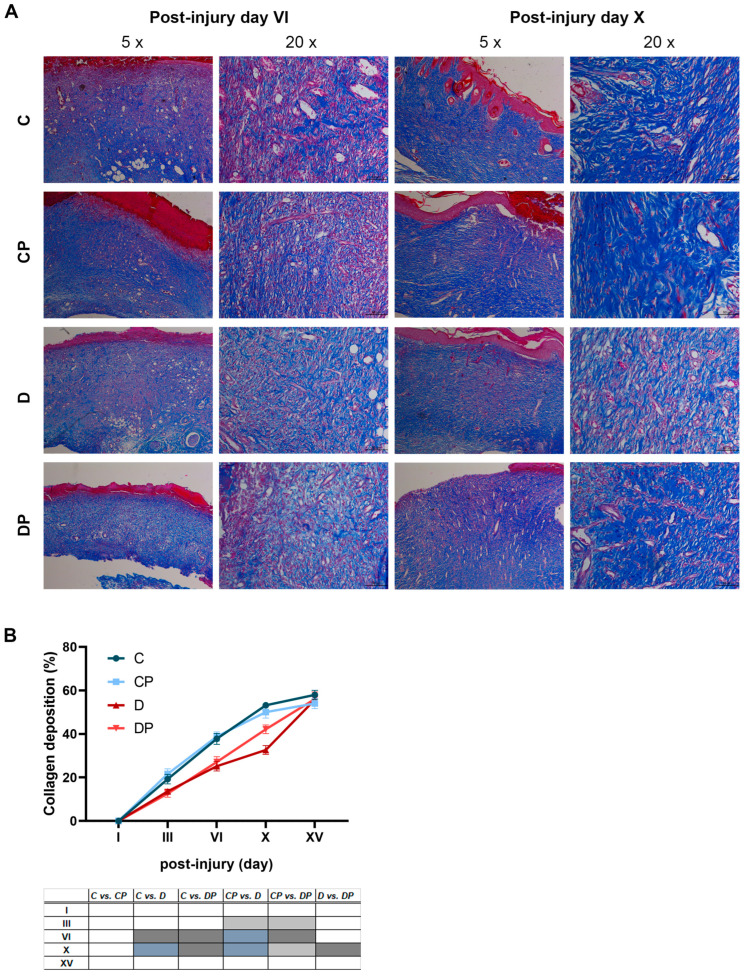
**The effect of PAW treatment on collagen deposition during wound healing in control and diabetic rats**. (**A**) Masson’s trichrome staining of wounds with or without PAW treatment in control and diabetic rats at days VI and X (magnifications 5× and 20×). (**B**) ImageJ software quantification of collagen (blue staining) expressed as % area within the sections. C–Control non-diabetic group (*n* = 3); CP–PAW-treated control non-diabetic group (*n* = 3); D–diabetic group (*n* = 3); DP–PAW-treated diabetic group (*n* = 3). Values are expressed as means of % area ± SEM, and the results of the ordinary two-way ANOVA and Tukey’s post hoc tests are presented in the tables below the graphs: light grey *p* ≤ 0.05; dark grey *p* ≤ 0.01; blue *p* ≤ 0.001.

Considering that collagen is a key component of ECM and plays an important role in regulating the different phases of wound healing, we wanted to investigate whether PAW treatment leads to changes in collagen deposition. The blue colour obtained after Masson’s trichrome staining showed a gradual increase in collagen deposition during wound healing from day VI to day X after injury, with better-organized collagen fibres that were more pronounced in both control groups compared to the diabetic groups (Figure 4A). Quantification of these results confirmed a higher percentage of the collagen content of the measured area in the C and CP groups compared to the diabetic groups at days III, VI and X. Specifically, the collagen contents on days III, VI and X were as follows: C (19.2%, 37.8%, 53.2%), CP (21.6%, 38.8%, 50%), D (13.6%, 25.2%, 32.7%) and DP (12.6%, 27.1%, 42.2%), respectively (Figure 4B). Furthermore, PAW treatment of the diabetic wounds increased collagen deposition by almost 10% on day X post-injury compared to untreated diabetic rats. Since collagen deposition is impaired in the wounds of diabetic rats, the increase in collagen content in the DP group suggests a positive effect of PAW on the wound healing process.

### 2.4. The Effect of PAW Treatment on Gene Expression

**Figure 5:**
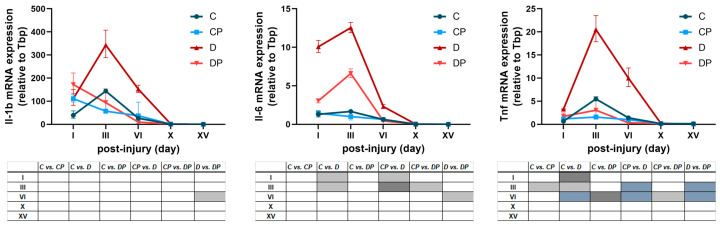

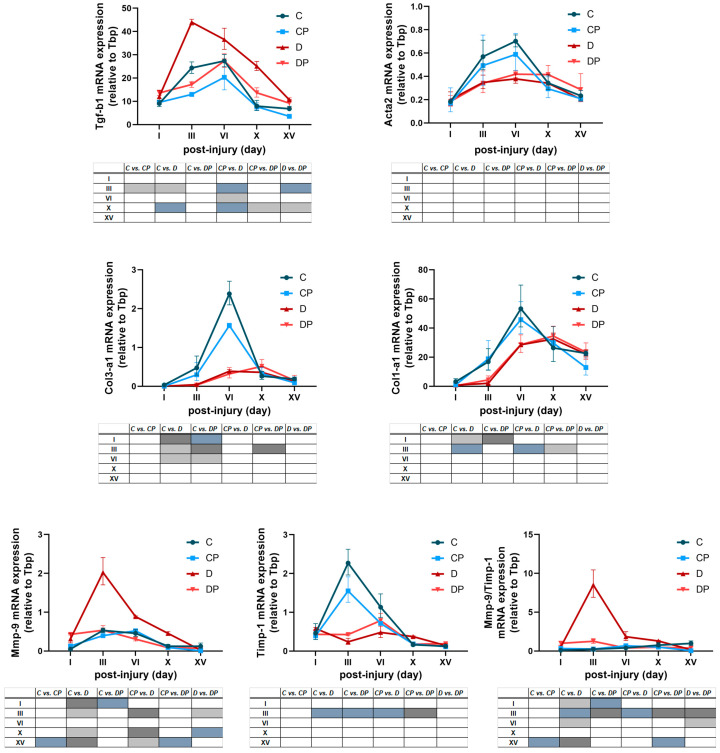
The effect of PAW treatment on the mRNA expression profile of selected genes in wound tissue from control and diabetic rats. C–Control non-diabetic group (*n* = 3); CP–PAW-treated control non-diabetic group (*n* = 3); D–diabetic group (*n* = 3); DP–PAW-treated diabetic group (*n* = 3). mRNA levels are presented relative to *Tbp* and are expressed as means ± SEM. The results of the ordinary two-way ANOVA and Tukey’s post hoc tests are presented in the tables below the graphs: light grey *p* ≤ 0.05; dark grey *p* ≤ 0.01; blue *p* ≤ 0.001.

The first group of genes whose expression was analyzed involved genes that play a role in the inflammatory phase of wound healing (Figure 5). PAW treatment altered the kinetics of the *Il-1β* expression with the highest value on day I and induced a gradual decrease thereafter, while *Il-1β* expression reached its maximum on day III in both non-treated groups. The *Il-1β* mRNA level was the highest in the diabetic group on days III and VI, while PAW treatment decreased it to a control level and reached a significant difference between groups D and DP on day VI after injury. The level of *Il-6* and *Tnf* mRNA was highest in the diabetic group, while the PAW treatment of diabetics significantly reduced the expression level of *Il-6* on day VI and of *Tnf* on days III and VI. Genes that are mainly involved in the proliferative phase of wound healing were tested to investigate whether changes in their expression were responsible for the observed differences in collagen deposition. The mRNA expression level of *Tgf-β1* remained elevated throughout the healing process, peaking on day III in the D group and on day VI in the other groups. From day III to X, PAW treatment decreased the expression of *Tgf-β1* mRNA in the diabetic group to the control level compared to the untreated diabetic group. The mRNA expression level of alpha-SMA (*Acta2*), a marker of activated fibroblasts (myofibroblasts), was higher in the control groups compared to the diabetic groups, but no significant changes in expression were detected after PAW treatment. In addition, the mRNA expression of *Col3-α1* and *Col1-α1* was higher in the control groups than in the diabetic groups, with no difference in their expression being seen between the D and DP groups. The kinetics of the mRNA expression of two collagen genes revealed more prominent and prolonged expression of *Col1-α1* mRNA after the fifteenth day, consistent with the switch from collagen III to collagen I in the later stages of wound healing.

The composition of the ECM depends on the balance between ECM synthesis and degradation, which depends on the expression of genes involved in the remodelling phase. *Mmp-9* and its tissue-specific inhibitor *Timp-1* play an important role in regulating the degradation and deposition of the extracellular matrix. Our results showed that PAW treatment had no effect on the mRNA expression of *Mmp-9* and *Timp-1* in control animals, except on day XV after injury, when the mRNA expression of *Mmp-9* was increased in the treated group. Compared to all other groups, the highest *Mmp-9* mRNA levels and the lowest *Timp-1* mRNA levels were observed in the diabetes group on days III, VI and X. *Mmp-9* mRNA decreased significantly in PAW-treated diabetic animals compared to untreated diabetic animals on days III and X. Accordingly, PAW treatment affected the *Mmp-9*/*Timp-1* ratio, which was 6.8- and 4.7-fold lower in the DP group compared to the D group on days III and VI, respectively. This result provides a possible explanation for the observed increase in the amount of collagen after the PAW treatment of diabetic animals.

## 3. Discussion

The concept of wound healing as a redox-driven process is well documented and widely recognized. RONS act as secondary messengers and play an important role in every phase of wound healing [34,35]. Therefore, CAP as a source of various RONS, applied in a spatially and temporally controlled manner, and its utilization for wound healing is a rational choice. However, excessive production of RONS and their increased and continuous presence leads to oxidative stress and impaired wound healing in diabetic patients [36]. Considering that only an exact balance of RONS levels leads to an improvement in wound healing, it is of utmost importance to determine the effect of plasma treatment on wound healing in diabetes, which is characterized by a redox imbalance.

Treatment of diabetic foot ulcers with CAP has shown moderate to significant improvement in healing in a few randomized clinical trials, leading to a faster reduction in the wound area and a decrease in the bacterial load [32,37,38,39]. Amini et al. reported reduced cytokine levels after CAP application, suggesting an antibacterial and anti-inflammatory effect of CAP [32]. In addition to a reduction in wound areas and accelerated healing after the application of CAP [40,41,42], animal studies showed faster re-epithelialization, improved collagen deposition and reduced inflammation [41,43]. Despite the undeniable benefits of CAP in the treatment of diabetic wounds, CAP systems are impractical as their application requires hospitalization. More recently, PAW has emerged as a potential solution capable of inactivating bacteria that commonly infect wounds [27,44] and improving wound healing in a mouse model with full-thickness wounds [27,45]. If the plasma is in contact with the water sample, the composition and concentration of RONS in PAW depends on the type of plasma source and the plasma parameters. Short-lived RONS created in the gas-phase plasma are deposited in the sample through the plasma/liquid interface and converted into long-lived RONS (H_2_O_2_, NOx-, etc.) [24]. Due to these properties, PAW can be stored for several days (months if frozen) and easily transported. The results presented in this paper extend the potential application of PAW in the treatment of diabetic wounds, as we found that twice-daily treatment with PAW accelerates re-epithelialization and improves wound healing.

Diabetic wounds appear to be stalled in the inflammatory phase and unable to move towards wound resolution [14]. Persistently elevated levels of proinflammatory cytokines such as IL-6, IL-1β, IL-8 and TNFα have been found in the wound bed fluids of patients with diabetic foot ulcers [32,46]. In addition to the persistence of increased numbers of neutrophils and macrophages in the wound bed of diabetic patients, the continuous production of proinflammatory cytokines results from the synergistic effect of hyperglycemia and hypoxia [47,48]. The results presented here indicate the strong anti-inflammatory potential of PAW in the treatment of diabetic wounds. Treatment with PAW reduced the number of inflammatory cells, including neutrophils and macrophages in the wound bed, and decreased the expression of *Il-1β*, *Il-6* and *Tnf* in diabetic rats. Similar results were obtained in a clinical study on the effects of CAP on diabetic foot ulcers in which it was found that the concentration of IL-1, IL-8 and TNFα in the wound bed decreased after CAP treatment [32]. PAW treatment of full-thickness wounds in control mice reduced the number of inflammatory cells [27] and decreased the expression levels of Il-1β and Il-6 [45]. Interestingly, on the first day of PAW treatment, the authors found higher levels of these two cytokines compared to untreated control samples, indicating the potential of PAW to induce a rapid inflammatory response [45]. In rat wound and burn models, significantly elevated levels of the proinflammatory factors Tnf and Il-1β were also detected in wound tissue 24 hours [49] and 48 hours after CAP treatment, with successive decreases in comparison to the control levels [50]. In our experiment, a comparable effect of PAW was observed on the first day of treatment, leading to an inflammatory burst followed by a subsequent decline in the inflammatory response. Namely, PAW/CAP treatment elicits a strong and rapid inflammatory response, enhancing the clearing of debris and preventing infection, which is particularly important for diabetic patients. At later time points, all inflammatory markers are lower in animals treated with PAW compared to untreated diabetic animals. Therefore, PAW treatment affects the inflammatory phase in diabetic animals in a specific two-step manner. The observed profiles of inflammatory markers indicate the strong anti-inflammatory potential of PAW, which allows for a resolution of the inflammatory phase and a transition to the proliferative phase, thus improving diabetic wound healing.

During the proliferative phase, epithelialization occurs through proliferation and the migration of keratinocytes, which has been shown to be promoted by CAP treatment [51,52,53]. Our results indicate that the PAW treatment of diabetic wounds leads to an improvement in epithelialization that is not different from epithelialization in control animals. Quantification of collagen deposition clearly discriminated control and diabetic animals, regardless of PAW treatment, except for PAW-treated diabetics, where a statistically significant increase in collagen deposition was observed on day X compared to the untreated diabetic group. Gene expression analysis revealed that diabetes drastically reduced the expression of two examined collagen genes and that the observed improvement in collagen deposition after PAW treatment was not due to increased expression of *Col3-α1* or *Col1-α1*, as their levels were found to be determined by hyperglycaemia rather than by treatment with PAW. Our results are consistent with the findings of another study that showed that plasma treatment does not affect collagen synthesis [49], suggesting that plasma treatments have limited effects on ECM deposition. However, collagen deposition depends on the balance between ECM formation and degradation by matrix metalloproteinases (MMPs). The activity of MMPs is regulated by the tissue inhibitors of metalloproteinases (TIMPs), and the MMP/TIMP ratio is one of the predictive biomarkers in chronic wound healing [2]. High MMPs concentrations and low TIMPs concentrations are typical features of diabetic wounds, which favour the degradation of the ECM and thus the impairment of wound healing [54]. We found that PAW significantly reduced the expression of *Mmp-9* in diabetic rats and consequently lowered the *Mmp-9*/*Timp-1* ratio to the level of the control groups. A possible explanation for the observed effect of PAW could be the differential regulation of Mmp9 and Timp1 expression by *Tnf* and *Il-1β*. Of the cytokines involved in MMPs regulation, *Tnf-α* has been shown to significantly upregulate *Mmp-9* protein in a variety of cells while suppressing the expression of *Timp-1* [55]. The upregulation of MMP-9 and downregulation of TIMP-1 was also the result of the presence of IL-1β [56]. Therefore, the observed decrease in the expression of *Mmp-9* and the decreased *Mmp-9*/*Timp-1* ratio may be a direct result of the dramatic reduction in the expression of *Tnf* and *Il-1β* in the PAW group compared to the diabetic group. Considering that MMPs can degrade and deplete growth factors and cytokines in addition to ECM proteolysis [57], the ability of PAW to reduce Mmp-9 expression may be another important factor in restoring the disturbed balance in non-healing diabetic wounds.

As we aimed to record wound healing at several time points, the number of animals per group has been minimized in accordance with ethical issues. Therefore, some effects could be underestimated due to the lack of statistical significance, which could not be reached. Nevertheless, important conclusions resulted from this study. Besides the antimicrobial activity that was documented elsewhere [27], we were able to show that PAW promotes diabetic wound healing by two additional mechanisms. PAW treatment influences the kinetics of the inflammatory response in diabetic wounds by triggering a rapid inflammatory burst followed by a decrease in the inflammatory response. In addition, PAW treatment affects the deposition and remodelling of collagen, and probably other ECM components, by decreasing the expression of *Mmp-9*. Our results suggest that PAW treatment has great potential in regard to accelerating the healing of diabetic wounds and contributing to the understanding of the molecular mechanisms responsible for the beneficial effect of PAW, enabling the development of improved strategies for the treatment of diabetic wounds.

## 4. Materials and Methods

### 4.1. CAP Treatments of Distilled Water–PAW Preparation

**Figure 6:**
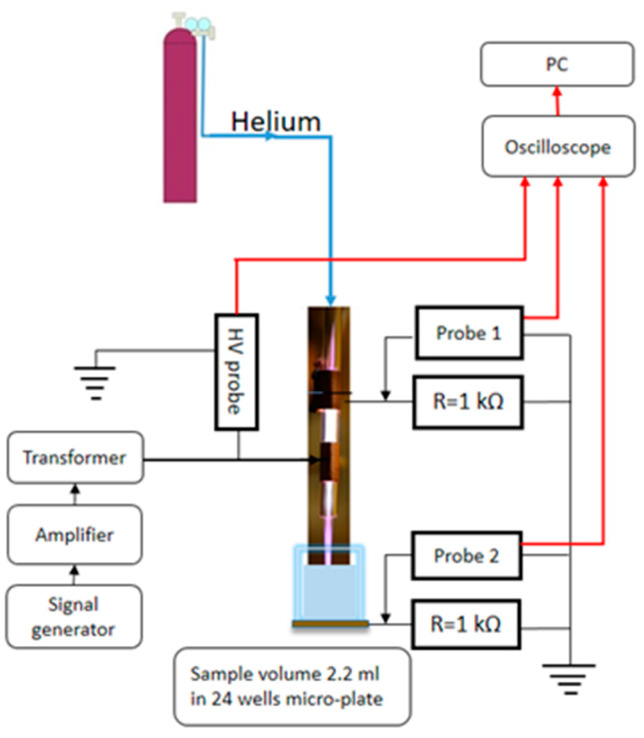
Schematic diagram of the DBD-type atmospheric pressure plasma jet system used for water treatments.

Figure 6 illustrates the Dielectric Barrier Discharge (DBD) type of atmospheric pressure plasma jet for treatments of distilled water. The DBD plasma jet consists of a glass tube with two 15 mm wide copper electrodes wrapped around the tube, and the gap between them is 5 mm. The electrode closer to the end of the tube was powered by a high-voltage sine wave at 80 kHz. The working gas was helium at 2 slm. The distance between the tube of the plasma jet and the surface of the sample was 5 mm. An amount of 2.2 mL of distilled water was treated in 24-well plates. The power transmitted to the plasma in contact with the sample was 0.5 W, and the treatment time was 10 min. After plasma treatment, colorimetric methods were used to determine the quantity of hydrogen peroxide (8 mg/L), nitrate (2 mg/L) and nitrite (0.2 mg/L). The concentrations of the measured RONS in PAW obtained in different treatments were highly reproducible. The pH of the PAW was not changed with respect to the distilled water (pH = 7).

### 4.2. Wound Healing Model

The study was approved by the Veterinary Administration, Ministry of Agriculture, Forestry and Water Management, Republic of Serbia (No. 323-07-04837/2021-05). Sixty male Wistar rats (200–250 g) were randomly assigned to four groups (*n* = 15 in each group): a control non-diabetic group (C), a PAW-treated control non-diabetic group (CP), a diabetic group (D), and a PAW-treated diabetic group (DP). Diabetes type 1 was induced by intraperitoneal injection of streptozotocin (Sigma, St. Louis, MO, USA) dissolved in citrate buffer (50 mg/kg) for five consecutive days while the control animals received citrate buffer. Two weeks after the streptozotocin injection, the blood glucose level was measured using a glucometer to confirm the establishment of diabetes. All animals were anesthetized by intravenous administration of Zoletil 100 (75 mg/kg) (Virbac, Suffolk, UK) and local subcutaneous administration of lidocaine hydrochloride (0.7%). Two full-thickness excisions were created on both sides of the posterior dorsal region with a 9 mm biopsy punch under sterilized conditions. Animals were caged individually, and, for the first five days, they received ketoprofen (5 mg/kg) (Kela, Hoogstraten, Belgium) for analgesia. From day 0, animals in the CP and DP groups received PAW treatment (300 µL − three applications from a spray bottle) twice a day at the same time during the whole experiment, while the C and D groups were treated with water. An equal number of animals (*n* = 3) were checked for blood glucose levels and euthanized on days I, III, VI, X and XV to perform further analyses.

### 4.3. Measurement of the Wound Closure Area

Photos of the wounds were taken on the day the wounds were made (day 0) and every time before euthanasia. The ruler was positioned next to the wounds as a scale bar. The wound closure area was calculated as the ratio of the current surface wound area to the initial surface wound area measured by ImageJ software (version 1.53t, National Institutes of Health, Bethesda, MA, USA) and expressed as a percentage.

### 4.4. Histological Analysis

One wound from each animal was fixed in 4% formaldehyde and mounted into paraffin blocks. A series of 7 µm thick sections from the middle of the wound were stained with haematoxylin–eosin (H&E) and Masson’s trichrome stain. The sections stained with H&E were photographed at 2.5× magnification and used for the determination of epidermal, dermal and total wound closure, as previously described [49]. Quantification of the inflammatory cells was performed by ImageJ from the ROI of 2592 × 450 px, acquired from six different section fields per animal, photographed at 40× magnification. Collagen deposition was measured on sections stained with Masson’s trichrome stain by ImageJ at four micrographs per animal obtained at 20× (ROI of 1024 × 768 px). The percentage of blue staining (collagen) was quantified by the Colour Deconvolution plug-in, with a manually defined threshold and vectors for each stain, and expressed as % area.

### 4.5. Determination of Myeloperoxidase (MPO) and N-acetyl-b-D-glycosaminidase (NAG) Activities

For protein isolation, 50 mg of wound tissue was used. The tissue was homogenated four times for 15 s in 1.5 mL of buffer (0.2 M NaH_2_PO_4_, 0.2 M Na_2_HPO_4_, 0.5% CTAB, pH 5.8) by using an Ultra-Turrax homogenizer (IKA, Staufen, Germany). The suspensions were freeze-thawed three times in liquid nitrogen, sonified three times for 10 s at 40 kHz using an ultrahomogenizer Sonopuls (Bandelin, Berlin, Germany) and centrifuged at full speed for 20 min at 4 °C. Assays were performed with 25 µL of the undiluted sample, as previously described [58]. Reactions that contained only the dissolver and without the substrate were used for normalization in both assays. Hydrolysis of the substrate was determined by measuring the colour absorption at 450 nm for the MPO assay and 400 nm for the NAG assay on a Synergy H1 microplate reader (Agilent BioTek, Winooski, VT, USA), and the enzymatic activity was expressed as A/µg of proteins.

### 4.6. Analysis of mRNA Expression by Quantitative Real-Time PCR (qPCR)

Total RNA extraction was performed with 30 mg of wound tissue with the use of an AllPrep DNA/RNA/miRNA Universal Kit (Qiagen, Hilden, Germany) according to the manufacturer’s instructions. Samples were immersed in RLT buffer with 1% β-Mercaptoethanol and 0.5% Reagent X with one 5mm stainless-steel bead (Qiagen), then disrupted and homogenized in a FastPrep-24 5G instrument (MP Biomedicals) for 40 s at 6 m/s with a pause of 5 min between three successive cycles. Reverse transcription was carried out with the RevertAid First Strand cDNA Synthesis Kit (Fermentas, Burlington, ON, Canada) on 2 μg of DNAse I-treated RNA using a mix of random hexamer and oligo(dT) primers. The qPCR was carried out in a final volume of 10 µL containing 50 ng of extracted RNA and 500 nM of each primer using the Maxima SYBR Green/ROX qPCR Master Mix (Fermentas) on a QuantStudio 3 Real-Time PCR system (Applied Biosystems, Carlsbad, CA, USA). The thermal cycles used were as follows: initial denaturation at 95 °C/10 min and 40 cycles of two-step PCR at 95 °C/15 s and 60 °C/60 s. Relative quantification of gene expression was determined using the 2-dCt method, with *Tbp* acting as the internal reference gene. A negative control was included in each array as well as two inter-run calibrators (control samples from intact skin and a mix of all samples in equal volume). Statistical tests were performed using log2 transformed data and mean values, and error bars were back-transformed to a linear scale for graphs. The primer sequences are listed in Table 1.

### 4.7. Statistical Analysis

A statistical analysis was performed using GraphPad Prism (version 8.0.2, GraphPad Software Inc., San Diego, CA, USA). A Shapiro–Wilk test was used to determine whether samples followed a normal distribution. For the comparison between groups, an ordinary two-way ANOVA was applied, followed by a Tukey’s test, and the null hypothesis was rejected at *p* ≤ 0.05. The experiments were performed in triplicate, and the data were expressed as mean ± SEM. The calculated standardized effect size (Cohen’s d) and the unstandardized measure of the effect size (Mean Difference and 95% CI) are listed in Table S1 in the Supplementary Materials.

## Figures and Tables

**Table 1:** Sequences of primers used for a real-time quantitative PCR analysis of gene expression.

Name		5′-3′ Sequence
*Il-1b*	fw	AGCAGCTTTCGACAGTGAGG
	rev	CTCCACGGGCAAGACATAGG
*Il-6*	fw	GTTTCTCTCCGCAAGAGACTT
	rev	ATACTGGTCTGTTGTGGGTGG
*Tnf*	fw	GCCACCACGCTCTTCTGTCT
	rev	CGCTTGGTGGTTTGCTACGAC
*Tgf-b1*	fw	CAGAACCCCCATTGCTGTCC
	rev	CCCTGTATTCCGTCTCCTTGG
*Acta2*	fw	ATCCGACCTTGCTAACGGAG
	rev	AGAGTCCAGCACAATACCAGTTG
*Col3-a1*	fw	AATGGGTGGCTATCCTGGAC
	rev	GGGTCTTCCTGACTCTCCATC
*Col1-a1*	fw	CACTGCAAGAACAGCGTAGC
	rev	AAGTTCCGGTGTGACTCGTG
*Mmp-9*	fw	GGGAACGTATCTGGAAATTCGAC
	rev	GTTGTGGAAACTCACACGCC
*Timp-1*	fw	TCTGCAACTCGGACCTGGTTAT
	rev	AAACTCCTCGCTGCGGTTCT
*Tbp*	fw	CTATGACCCCTATCACTCCTGC
	rev	GCAGTTGTTCGTGGCTCTCTTA

## Data Availability

The original contributions presented in this study are included in the article/Supplementary Material. Further inquiries can be directed to the corresponding authors.

## Supplementary Materials

The following supporting information can be downloaded at: www.mdpi.com/article/10.3390/ijms26031265/s1.
